# Men ask more questions than women at a scientific conference

**DOI:** 10.1371/journal.pone.0185534

**Published:** 2017-10-16

**Authors:** Amy Hinsley, William J. Sutherland, Alison Johnston

**Affiliations:** 1 Durrell Institute of Conservation and Ecology, School of Anthropology and Conservation, University of Kent, Kent, United Kingdom; 2 Department of Zoology, University of Oxford, Oxford, United Kingdom; 3 Conservation Science Group, Department of Zoology, Cambridge University, Cambridge, United Kingdom; 4 The British Trust for Ornithology, The Nunnery, Thetford, United Kingdom; 5 Cornell Lab of Ornithology, Cornell University, Ithaca, United States of America; Universitatsklinikum Tubingen, GERMANY

## Abstract

Gender inequity in science and academia, especially in senior positions, is a recognised problem. The reasons are poorly understood, but include the persistence of historical gender ratios, discrimination and other factors, including gender-based behavioural differences. We studied participation in a professional context by observing question-asking behaviour at a large international conference with a clear equality code of conduct that prohibited any form of discrimination. Accounting for audience gender ratio, male attendees asked 1.8 questions for each question asked by a female attendee. Amongst only younger researchers, male attendees also asked 1.8 questions per female question, suggesting the pattern cannot be attributed to the temporary problem of demographic inertia. We link our findings to the ‘chilly’ climate for women in STEM, including wider experiences of discrimination likely encountered by women throughout their education and careers. We call for a broader and coordinated approach to understanding and addressing the barriers to women and other under-represented groups. We encourage the scientific community to recognise the context in which these gender differences occur, and evaluate and develop methods to support full participation from all attendees.

## Introduction

Gender imbalances in science, technology, engineering and mathematics (STEM) careers have been widely recognised, with women particularly underrepresented at senior levels [1; 2]. This so-called ‘leaky pipeline’ effect is often particularly apparent in academia, where even subjects with gender parity (or even a majority of women) at undergraduate and postgraduate levels see declining proportions of women with increasing seniority, and very few in senior positions [3]. There is debate over whether this is an artefact of demographic inertia (senior positions represent past gender ratios) [3], but there is also evidence of a disproportionate lack of women in some of the key activities underpinning academic career progression, suggesting inertia may not be the sole cause. These activities include securing prestigious grants [4], acting as the final or corresponding author on peer-reviewed papers [5], patenting important findings [6] and participating in high-profile sessions at conferences [7; 8]. Attention is often paid to the role of discrimination and bias, both conscious and unconscious, in creating these differences (e.g. [8]).

Many efforts to tackle the gender imbalance in career activities have focussed on addressing the most visible (and potentially unconscious) forms of discrimination, including the introduction of double blind peer review [9], lists of female scientists for conference organisers (e.g. http://www.academia-net.org/), and codes of conduct for professional meetings (e.g. [10]). These measures are a positive step, but the problem is complex and further work is needed to identify and understand the roles of different underlying factors, to assist in addressing the problem [11]. Whilst there is evidence of bias against women, for example in the rating of research quality [12], in the peer-review process [13], or in the likelihood of invitation to speak on conference panels [7], this is not always found. For example, studies have found no evidence of gender bias in the outcome of journal peer-review decisions, even though gender-based patterns of publication behaviour differed [5; 14; 15]. These differences in behaviour point to another explanation, that the problem is linked to gender-aligned differences in behaviour that favour the success of men [16]. One established theory that combines both of these explanations proposes that subtle and often unconscious biases create so-called ‘chilly’ academic environments [17] that give subtle cues to women that they do not belong [18].

In this paper we focus on examining gender differences and potential causes in a relatively understudied setting: participation in conference question and answer sessions. Conferences are important events that allow scientists to establish themselves as an expert in their subject, connect with potential collaborators, and discuss emerging work in their field. However, in spite of its importance to academic career progression, relatively little research has been conducted into patterns of conference participation [19]. In terms of gender differences at conferences, research has focussed on participation via presentations [7; 8; 20], with studies of question and answer sessions limited to the field of astronomy [21; 22]. In these studies women asked fewer questions, a finding attributed to demographic inertia, due to the proposed increased likelihood of question-asking by senior scientists [21; 22]. However, this suggested ‘age-effect’ was not tested in either study, although evidence from the wider literature on gender and participation suggests that this finding could be part of a wider pattern.

Whilst question-asking has been little studied at conferences, the topic of gender differences in ‘speaking up’ and participation in a classroom setting has been of great interest to the academic community for several decades. Reviews of published studies have found that overall, women participate less often and with lower confidence in the classroom [23]. For example, a meta-analysis of 81 studies of gender-based differences in classroom interactions found that boys participated significantly more than girls [24]. This is supported by more recent studies, which have found that males tend to dominate face-to-face discussions in both the school [25] and university classroom [23]. However, this is not simply a case of innate differences in gender behaviour as these patterns have been shown to increase with age. In schools, teacher-pupil interactions are broadly equal in lower year groups, gradually becoming permanently skewed towards boys after the age of nine [24] or 13 [26], with male 13 to 14 year-old students 2.5 times more comfortable asking questions than females [27].

The decline in participation by female students is often linked to self-esteem (e.g. [27]), supported by some studies showing that women have lower self-esteem than men at all ages [28]. Van Der Meij [29] found that self-esteem and question-asking have a complex relationship, with both very high and low self-esteem linked to a desire for self-preservation. This self-preservation may be in the form of a desire to appear less aggressive in front of teachers or peers, or a lack of confidence in their ability to pose an appropriate question [26]. Confidence in academic abilities can be undermined in women by subtly biased teacher and peer behaviour in the classroom itself [30] and outside it [17], and biases in interaction and feedback in an academic setting can result in students becoming passive in the classroom [31]. For example, younger girls are slightly more likely than boys to raise their hands to ask a question but are less likely to be allowed to do so by teachers [24].

**Fig 1:**
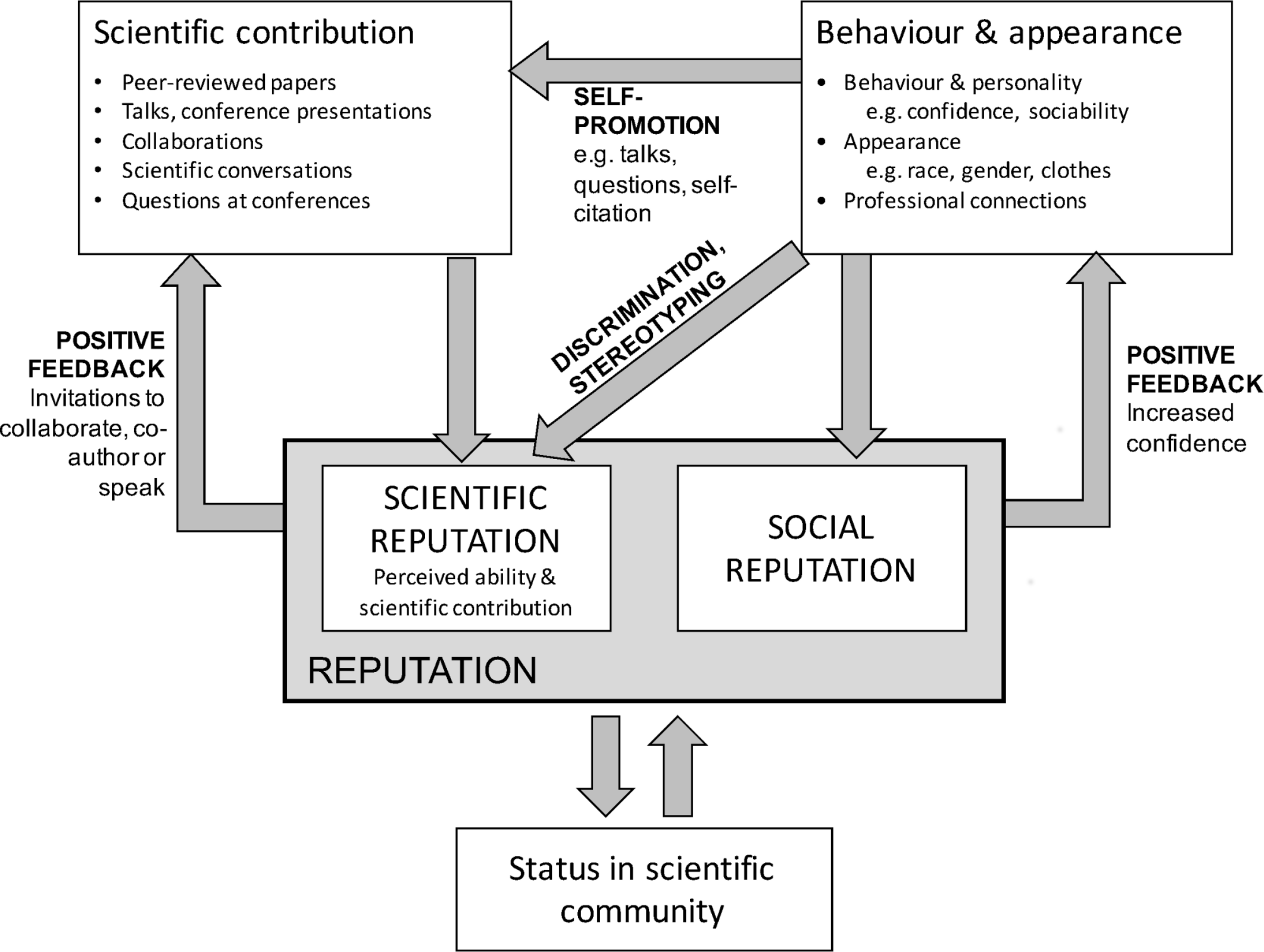
The reputation model. This assumes people have two properties of interest: their *Scientific contribution*, which can be considered as the type of information that people add to their cv and their *Behaviour and appearance*. The *Scientific contribution* is partly determined by the behaviour through the degree of self-promotion, such as volunteering to give talks. We divide reputation into *Scientific reputation*, how good a scientist someone is considered to be, which is determined by a combination of the *Scientific contribution* and *Behaviour and appearance* through discrimination and stereotyping. The balance of these is likely to differ between contexts, for example assessing applicant for a job may be based largely on comparing CVs, while deciding who to invite to a workshop may be a less evidence-based process for which impressions play a greater role. There is also the *Social reputation*, for example is how enjoyable company a person is perceived to be. By *Status in the scientific community* we are thinking of formal positions, such as invitations to be an editor or positions within academic organisations. Invitations will consider both their scientific and social reputations. Status will feedback into reputation. Finally, there are positive feedback looks between reputation and Scientific contribution, for example through invitations to join projects or good students or postdocs being keener to join the group, as well as between reputation and behaviours, for example by being more confident.

Fig 1 shows the Reputation model, which we propose as a means of considering many of the ways in which factors such as appearance or behaviour may influence scientific outputs and position in the community. In this model behaviour and appearance can have four routes to affect science and status. Firstly, it may determine whether people show self-promotion, for example by offering to give talks, asking questions or approaching others at meetings. Secondly, it may affect scientific reputation. In this model scientific reputation is a combination of actual achievements and prejudice resulting from assessment of behaviour and appearance. Thirdly it may affect social reputation, which, for example, is whether a person is considered likeable or not. This may affect whether is invited to events. The reputation affects status in the community, for example whether awarded prizes, invited to be on editorial boards etc. This feeds back onto reputation. Finally, reputation has feedback leading to both increased scientific contribution (for example invitations to give talks or collaborate) and also behaviour (for example greater confidence).

This pattern of lower participation from women can also be considered within this theoretical framework. Scientific reputation is obviously important although it is clearly not one dimensional, for example individuals may be considered especially strong in generating ideas, experimental practice, analysis or in making penetrating comments. High scientific reputation, as with other positive feedback, may enhance personal wellbeing as well as potentially leading to offers to participate in meetings, or suggestions to collaborate. As an example of its importance, Göktepe-Hulten and Mahagaonkar [32] showed that patenting activities of scientists were better explained by enhancing scientific reputations than direct financial gain.

However, as demonstrated in the Reputation model, scientific reputation may be poorly related to actual contributions to the field, with some obtaining high credit due to the promotion by themselves or followers [33]. In addition, an individual’s perception of someone else’s scientific reputation may result from a direct assessment of their science, from the comments of others or from perceptions during encounters. Although the literature concentrates on the discussion of metrics (e.g. [34]), we suggest that scientific reputation is more complex and often not solely based on rigorous measures but on a combination of different factors. These may include informal discussion and experiences of another person, meaning that how a person appears or behaves may contribute towards reputation, especially for those, such as early career researchers, for which there is little other evidence. In a similar vein, Wei and Stillwell [35] examined how profile images on Facebook affect perceptions of intelligence and showed that some features, such as whether wearing glasses or smiling, correlated with perceived intelligent but not actual intelligence. Participation in meetings is therefore likely to play a role in the assessment of scientific reputations. Reputation may then lead to invitations such as opportunities to collaborate, give talks, apply for positions etc and so influence the actual contribution made.

Within the context of this framework, here we hypothesise that women are less likely to participate in the question sessions at a large scientific conference, due to behavioural differences linked to external factors. We aim to address the question of whether women ask fewer questions than men in this context and to investigate the influence of factors other than gender-based behavioural differences, such as demographic inertia and discrimination by session chairs. Finally, we aim to discuss whether the likely causes of gender differences in behaviour in these settings are likely to be addressed by current efforts to tackle the gender imbalance in STEM fields. This is the first study of this scale to compare participatory behaviour of different genders at an international conference. Significant differences in behaviour would highlight that there are behavioural patterns aligned with gender (but not restricted to specific genders), that may be a symptom of and/or a contributing factor to, the continuing drop out of women at progressively higher levels of seniority in science.

## Methods

### Data collection

We observed sessions of talks at the International Congress for Conservation Biology and European Congress for Conservation Biology in Montpellier, France in August 2015. At four randomly selected times during the week we monitored up to 10 synchronised parallel sessions. Monitoring was conducted with a team of 14 voluntary observers for 34 of these 40 talk sessions. Each observer recorded the number of men and women in the audience (excluding themselves), the number of men and women sitting in the front half of the room, and the genders of the session chair(s), each speaker, and audience members selected to ask questions. For 20 sessions, the age of those asking questions was recorded in two categories: younger than 50, or 50 or older (based on a visual assessment). See Table 1 for a summary of the variables recorded and S1 File for further details of data collections methods. Six sessions were monitored with two independent observers and these results suggested good consistency between observers with the gender ratios in the room and the gender of people asking questions (see S1 File for further information). We note that these methods treat gender as a binary dichotomy, which is not the case, as many people do not identify with a single gender. In addition, the visual assessment of gender assumes that people identify as the gender they appear. This will not always be true, however we expect the cases in which the observers assigned the wrong gender to be relatively few in number and therefore not to bias the overall conclusions. The data are available in S2 File.

**Table 1:** Parameters recorded in each session.

Session	Talk	Question
Number of men (*n*_*m*_) and women (*n*_*f*_) in the room at the end of the first talk	Gender of speaker (*gen*_*speak*_) binary: 0 = female; 1 = male	Gender of person asking question (*gen*_*quest*_) binary: 0 = female; 1 = male
Number of men and women in the front half of the room		Age (younger/older than 50) of person asking question (*age*_*quest*_) binary: 0 = younger than 50; 1 = 50 or older
Gender of session chair(s) (*gen*_*chair*_) binary: 0 = female; 1 = male		

List of the variables recorded at the level of the session, talk, and question.

All work was carried out with the approval of the University of Kent’s School of Anthropology and Conservation Ethics Committee. We obtained permission from the conference organisers in advance, but delegates were not informed about the study, to ensure that results reflected the typical behaviour of chairs and conference participants. We ensured that all data from sessions were aggregated; no personal or identifying information was collected about any individual.

### Statistical analysis

We analysed question data from 31 sessions (three sessions were removed for incomplete data or the chair being aware of this study). Across the 31 sessions, we analysed 270 questions.

To test whether men asked significantly more questions than women, we used a binomial Generalized Estimating Equation (GEE) [36] with session ID as the repeated measure and an independent variance structure. Preliminary testing with a hierarchical model structure and session as a random effect estimated a random effect variance of zero, suggesting there were no detectable differences between sessions. In this case, GEE is a more conservative approach that maintains the non-independence of data points within sessions. The response variable in the GEE was whether each question was asked by a male or female audience member (*gen*_*quest*_; 1 = male; 0 = female), so there was one data point for each question asked. We used a logistic regression model [37] to estimate *p*, the probability that a question is asked by a male audience member. As the gender ratio of the audience determines the numbers of each gender that are available to ask questions, the logit-transform of the proportion of the audience that was male (*n*_*m*_*/(n*_*m*_*+n*_*f*_*)*) was included as an offset:

$$logit\;(\hat{p})=\beta_{0}+logit\;\left(\frac{n_{m}}{n_{m}+n_{f}}\right)$$ (1)

If men and women asked questions at the same rate, we would expect the intercept of the binomial model (*β*_*0*_) to be zero on the logit scale, which implies an equal rate of questioning by men and women after accounting with the offset for the proportion of men and women in the room. We formulated the data such that 1 = male question and 0 = female question, therefore a positive intercept suggests that men ask more questions than women and a negative intercept suggests the opposite.

We included gender of the session chair and the speaker as covariates and used "quasi-likelihood under the independence model criterion” (QIC) [38] to distinguish between models. These comparisons were necessarily run with a slightly smaller dataset with 24 sessions and 188 questions (excluding questions asked in a general discussion and excluding sessions that had no chair or both male and female chairs).

$$logit\;(\hat{p})=\beta_{0}+\beta_{1}{gen}_{chair}+\beta_{2}{gen}_{speak}+logit\;\left(\frac{n_{m}}{n_{m}+n_{f}}\right)$$ (2)

Eq 2 describes the estimate of the logit probability of the questioner being male (*p*).

Previously it has been hypothesised that men ask more questions because there are currently more men in senior positions and experienced people have greater confidence and often a wider knowledge. To test this hypothesis, we repeated the question analysis above, using only those questions asked by people in the younger age group category (less than 50). Only some observers recorded the age of the questioners, therefore this analysis was conducted with data from 20 sessions and included 123 questions.

We did not have information of the gender balance of young people in the audience as we considered achieving this would make our activities too conspicuous, therefore we used two separate approaches to estimating the gender balance of young people in the audiences. Firstly, we assumed the proportion of men and women was the same in the younger age group as all age groups. However if younger age groups are likely to have a greater proportion of women than older age groups, this will over-estimate the number of young men and under-estimate the number of young women. Secondly, we assumed that in each room 10% of participants were at least 50 years old and we assumed that 75% of those over 50 were male (based on estimates of gender balance of science A1/professor researchers within the EU [2]. We used these two assumptions to estimate an adjusted proportion of the younger (<50) age group that was female. We used the same GEE structure described in Eq 1 above with the gender of the questioner as a binomial response, the logit transform of the estimated proportion of younger men in the audience as the offset and the session ID as the repeated measure.

All models were carried out in the software R [39] using the package ‘geepack’ [40].

## Results

In the 34 sessions of talks the mean proportion of the audience that was female was 0.57. There was no significant difference in the proportion of men and women that chose to sit in the front half of the room (paired t-test: t = 0.354, df = 33, p = 0.726).

In total across all 31 sessions analysed for questions, there were an estimated 1487 female audience members and 1116 male audience members. There were 270 questions: 152 from men and 118 from women. Therefore, on average there were 0.08 questions asked by each female audience member and 0.14 questions asked by each male audience member.

**Fig 2:**
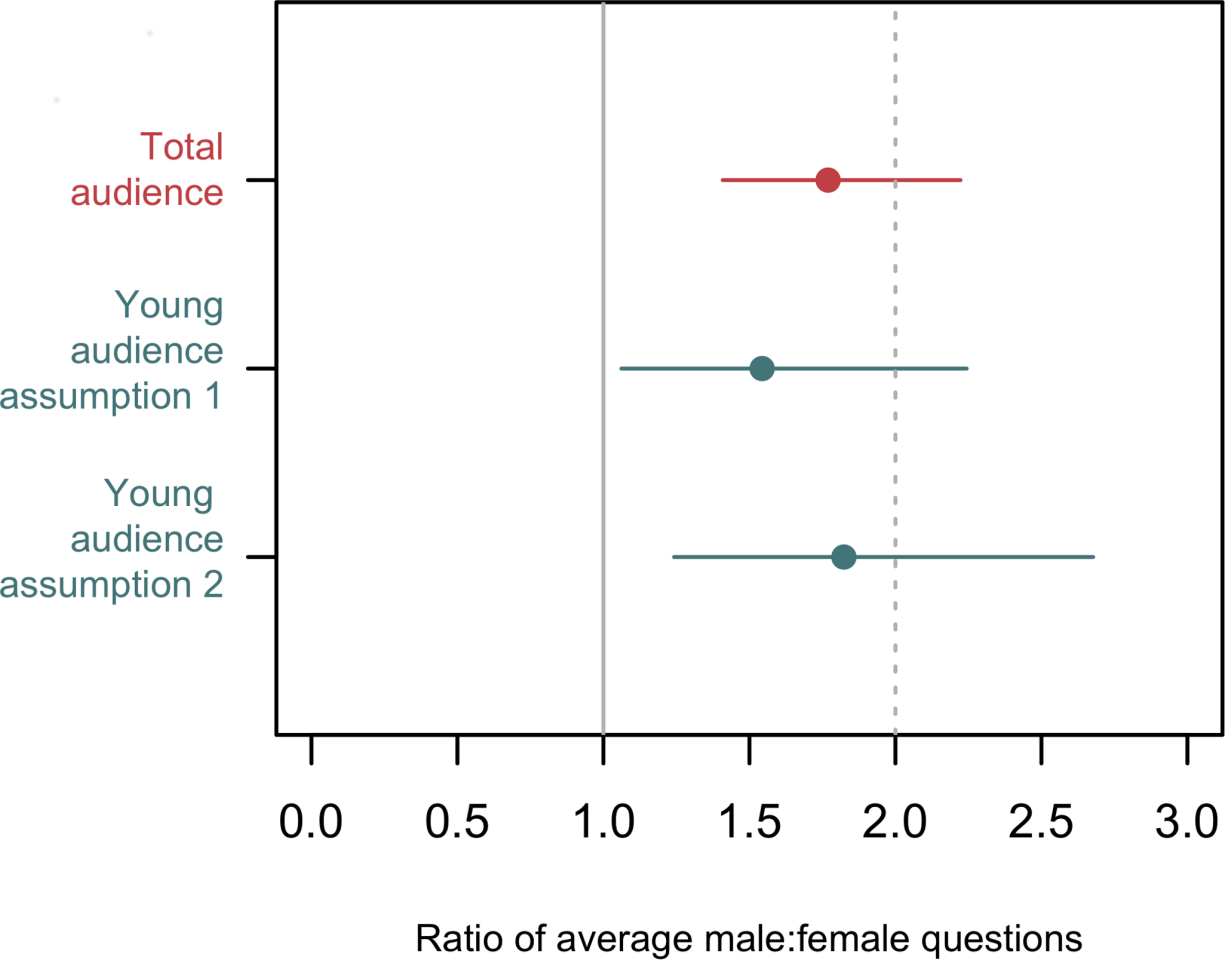
Estimated ratio of questions from male and female scientists. The estimated ratio of male:female question rate estimated from the overall model and the two models with only those people judged to be under 50 years old. The grey lines indicate the point at which male audience members ask the same number of questions as female audience members (solid grey line) and twice as many questions (dashed grey line). The “<50 model observed proportion” is the model with the offset using the first assumption–that proportion of the younger audience that is male is the same as the observed proportion of the entire audience. This is likely to be an overestimate of the proportion of men in the younger age group, and the estimated ratio shown here will likely be negatively biased as a result. The “<50 model adjusted proportion” is the model with the offset using the second assumptions and adjusting the estimated proportion men and women in the younger audience to account for the fact that more senior researchers are male.

After accounting for the gender balance in the audiences, men asked more questions than women, demonstrated by a positive intercept in the GEE model (intercept = 0.57; se = 0.12). Transforming this intercept back to the real scale, the estimated values suggest that in a hypothetical room with an even balance of men and women, 64% of questions would be asked by men (95% confidence interval 59%-69%). This point estimate suggests that men ask nearly two thirds of the questions and therefore for each question asked by a woman, each man asks on average 0.64/0.36 = 1.8 questions (95% CI 1.4–2.2) (Fig 2). There was very little evidence that chair gender and speaker gender improved the model fit (ΔQIC > 8 for the addition of each covariate and model weight 0.98 for the model with only an intercept; see S1 Table for details).

Even amongst the younger researchers, male participants asked significantly more questions than female participants. Using the first estimate of gender ratio, the model estimated that, in a room with an even gender balance, young men would ask 61% of the questions asked by younger researchers and that therefore younger male researchers ask 1.5 questions (95% CI: 1.1–2.2) for every question asked by younger female researchers. However, we expect that this model will produce a negatively biased estimate of the ratio of male:female questions, given the biased offset used. Using the second adjusted estimate of gender ratio, the model estimated that with an even gender balance, young men would ask 66% of the questions asked by younger researchers and that therefore younger male researchers ask 1.8 questions (95% CI: 1.2–2.7) for every question asked by younger female researchers (Fig 2).

We recorded only 36 instances of competition between at least two people to ask a question and 20 of these were between male and female audience members. In 12 of these 20 instances, a man was selected to ask the question. This ratio is similar to the overall bias in questions, however, the sample size is too small to draw any statistical conclusions. Nonetheless, this small sample suggests that there was not a substantial amount of competition to ask questions.

## Discussion

By observing question and answer sessions at a large international conference we show that, even accounting for the gender ratio of the audience, male researchers ask more questions than their female counterparts. This confirms previous findings and, for the first time, demonstrates that this pattern is seen in both younger and more senior researchers, and is therefore unlikely to be a result of demographic inertia.

Decades of research has shown a decline in classroom participation with age for female students [24], and our results provide evidence that women continue to participate less than men in professional academic settings. The subject of participation at scientific conferences is little-studied, although the importance of participation and ‘speaking up’ in professional meetings has been emphasised as key to career progression in other sectors [41]. When considering our reputation model (Fig 1), participation at academic conferences is likely to be important, as conferences provide opportunities to build reputations, establish and maintain professional networks, and develop international collaborations [42]. As self-promotion and behaviour are also important in building academic reputation [33], the behavioural differences we have described could contribute towards the underestimation of female scientists’ abilities [43], and their overall reputation, as has been suggested in the case reputational benefits of higher self-citation publication rate of males [44]. We also expect participation at an international conference is likely to be correlated with participation in other professional situations, therefore these observed differences in gender behaviour in question asking are important.

We found that on average a male researcher asks an estimated 1.8 questions for each 1 question asked by a female researcher. Two similar studies at astronomy conferences estimated that men asked more questions than women [21] and men asked 1.8 questions for every question asked by women Pritchard *et al*. [22]. However, these previous studies did not account for the gender ratio in each session, only the overall gender ratio at the conference. Therefore, these previous results could be biased if men and women attend different numbers of talks, or if the total number of questions varied with the gender balance of the session. These previous studies into gender differences in participation [21; 22] were also conducted with the knowledge of conference participants, which may have led to stereotype bias, the process by which knowledge of a stereotype can become self-fulfilling and impact performance [45]. Ours is therefore the first study to confirm that men ask significantly more questions than women, whilst accounting for the gender ratio of the session audience and without any potential for knowledge of the study to impact behaviour.

Given that even amongst the younger age bracket, men asked significantly more questions than women, our findings suggest that behavioural patterns are not only a result of ‘demographic inertia’, in which historical inequalities persist due to a lag time [3; 21; 22]. These results indicate more complex underlying reasons for differences in participation that may need to be addressed directly.

We observed differences in participation throughout our sample and also found no evidence that the gender of the session chair or speaker affected the number of questions from male and female audience members. This result is in contrast to Davenport *et al*. [21] who found that male session chairs had significantly more questions from male participants. Our findings may therefore suggest innate gender differences in participation or that male and female chairs both display the same bias towards male questioners. The small sample size from the instances in which participants competed to ask questions was too small to draw any conclusions about bias or discrimination. However, the lack of questions in which there was competition does suggest a limited impact of direct discrimination in this conference. Nevertheless, this type of discrimination over many years may lead to a long-term effect of women volunteering questions less often, eventually leading to this discrimination no longer being detectable due to lower participation by women.

Differences in participation in question sessions may be supported by other studies of conference participation that have found that women are more likely to apply to present their work in conference poster presentation sessions rather than oral presentations [7; 46]. Similarly, in business, underrepresentation of women on company boards has been attributed to women’s reluctance to participate and make themselves heard [41]. However in contrast to other studies [47], we did find that women and men were distributed equally across the front and back of the room, suggesting women were choosing to sit in similar locations to men.

Here, we do not assume that ‘male’ behaviour is correct and that women need to behave more like men; we recognise that there are other methods of participating at a conference and that the use of different communication strategies may need further research. We also reject the idea that our findings are linked to the women in our sample being less intelligent than the men, as the relationship between gender and intelligence is widely accepted to be complex [48]. Whilst some research has found that, after puberty, men have higher average intelligence scores than women [49], in other studies these differences between men and women are often found to be non-existent, highly variable, or attributed to external factors [48]. In addition, our sample was of predominantly post-graduate scientists at a professional conference, so we assume that attendees of both genders were at the same level. Rather, we suggest that the observed behavioural differences are important to understand in more detail, and cannot be separated from the wider context in which they occur. It is possible that our results reflect wider findings of greater competitive behaviour amongst men than women [50], and that men are using questions as a way of competing with others and showcasing their own knowledge and work. Whilst asking a question in and of itself may not always be of immediate tangible benefit to the questioner, as previously discussed it may serve to increase status and build academic reputation, which are likely to be important to career progression. In this study, we did not record the different types of questions asked, for example simple requests for information, criticisms of the presented work, or comments focussing on the questioner’s own research. We note that there could have been a difference in why men and women ask questions, and future work could investigate potential differences and links to competitiveness and reputation building. However, whether driven by competitiveness or other factors, the act of asking a question is linked to higher levels of self-confidence [26], with lower confidence linked to a desire for self-preservation that makes question asking less likely [29]. In many cases, current gender differences in behaviour are likely to be a long-term response to wider experiences of inequality that women may have faced [17; 51]. Therefore, our findings may reflect a lower level of self-confidence amongst female scientists, who are likely to have faced academic and professional barriers based on their gender that men have not. There are a number of documented examples of men having fewer barriers to academic career progression, for example, men have been found to be preferentially selected for jobs [52], have their research rated higher quality [12], and awarded grant funding by peer reviewers [4; 53]. There is also evidence that female scientists are more likely to have experienced harassment during fieldwork [54]. Whilst conducting our study, an observer was told by a male audience member that a young female speaker arriving on the podium to begin her talk “didn’t look like she knew much about science”. Although anecdotal, this interaction highlights the continued negative attitudes and discrimination that women face in professional situations.

Our findings of gender differences are of particular interest, as the conference in question had taken steps to address inequality and discrimination in its most visible forms, using a clear code of conduct [10]. It is critical to progressing gender equality that these measures continue; there is evidence from a wide variety of scientific fields that the inclusion of women on the selection committee for invited speakers has been shown to increase the number of female speakers invited [7; 8; 55]. This pattern was also observed at the conference that our research was conducted at but, in spite of broad measures to address gender imbalances, the proportion of female organizers and speakers has not increased overall [20]. We suggest that further targeted efforts may be needed to encourage participation in specific areas. For question-asking, behavioural studies have found that men increasingly dominate classroom discussion from older school ages [26], but women may be more likely to post on forums [23], and therefore Twitter questions may be a possible strategy to receive more questions from women.

We acknowledge that further work is needed to fully explore the issues surrounding gender and participation, and address some of the limitations of our study. In particular, further work to understand the underlying causes of the gender differences that we observed would be of benefit. For example, our observation methods were designed to ensure that attendees behaved normally, but future work could also carry out follow-up surveys with attendees to look at motivations for asking, or not asking, a question. In addition, we did not record the types of question asked, which may provide more information on the purpose of questioning (e.g. to demonstrate the questioner’s own knowledge, to provide criticism, or simply to request additional information) and how this might link to the gender of both the speaker and the questioner. The use of observation in our study also meant that we relied on gathering data on age and gender based on appearance, which may have led to some errors. In particular, we note that gender and appearance do not always align. Following on from this, we also note that we have treated gender as a binary dichotomy, and, we recognise that an increasing number of people do not identify with a single gender. These individuals, as well as other race demographics, are also likely to suffer discrimination and bias in academic science, however the evidence and sample sizes for these groups are extremely small. Here we examine women, who represent a larger proportion of the conservation biology community, as an example of how gender-aligned behaviour might be affected by and contribute to bias and discrimination.

We found that behavioural differences in gender exist in younger researchers, and may be affected by self-esteem, or discrimination in school or professional environments. Therefore, our findings support assertions that the issues surrounding gender imbalances in STEM and particularly in academia are complex and in need of better understanding [11]. However, the Davenport [21] and Pritchard [22] studies confirm that awareness alone is insufficient to remove the problem. There are an increasing number of organisations that have acknowledged the problem by aiming to eradicate discrimination and remove the influence of bias, including unconscious bias. Given the lower average self-esteem of women [28] perhaps caused by the biases in society and the academic communities, science should make concerted efforts to produce equal gender participation. There needs to be a broader and more coordinated approach to understanding and addressing the barriers to women and other underrepresented groups in STEM. For conferences in particular we encourage the scientific community to recognise the context in which these gender differences occur, and evaluate and develop different methods to support greater participation from all attendees.

## Supporting information

S1 Table Summary of model selection. Model selection table for Generalized Estimating Equations (GEE) with data from 24 sessions that included data on chair gender and speaker gender and either male or female sessions chair(s) (i.e. excluding sessions with no chair or both male and female chairs). After removing questions asked in a general discussion session, 188 questions were included in the model.
(DOCX)

S1 File Data collection and analysis. Further details of data collection and analysis methods.
(DOCX)

S2 File Questions dataset. Dataset used for the analysis. Columns include the session number, the gender of the chair, gender of the speaker, number of questions to that speaker from female audience members, number of questions to that speaker from: male audience members, female audience members under 50, male audience members under 50, the proportion of the audience that was male, the estimated and adjusted proportion of young audience members that were male.
(TXT)
