# Supplementary material for: Men ask more questions than women at a scientific conference
Source: PLoS One. 2017 Oct 16;12(10):e0185534. doi: 10.1371/journal.pone.0185534 (PMC5643049; doi:10.1371/journal.pone.0185534)
Supplement: S1 Table — Model selection table for Generalized Estimating Equations (GEE) with data from 24 sessions that included data on chair gender and speaker gender and either male or female sessions chair(s) (i.e. excluding sessions with no chair or both male and female chairs). After removing questions asked in a general discussion session, 188 questions were included in the model. (DOCX) [file pone.0185534.s001.docx]

**S1 Table. Summary of model selection.**

| Model | QIC | Δ QIC | Model weight |
| --- | --- | --- | --- |
| Intercept | 68.1 | 0 | 0.976 |
| Intercept + chair gender | 76.6 | 8.5 | 0.014 |
| Intercept + speaker gender | 77.2 | 9.2 | 0.010 |
| Intercept + chair gender + speaker gender | 85.5 | 17.4 | 0.000 |

Model selection table for Generalized Estimating Equations (GEE) with data from 24 sessions that included data on chair gender and speaker gender and either male or female sessions chair(s) (i.e. excluding sessions with no chair or both male and female chairs). After removing questions asked in a general discussion session, 188 questions were included in the model.
