# Supplementary material for: Men ask more questions than women at a scientific conference
Source: PLoS One. 2017 Oct 16;12(10):e0185534. doi: 10.1371/journal.pone.0185534 (PMC5643049; doi:10.1371/journal.pone.0185534)
Supplement: S1 File — Further details of data collection and analysis methods. (DOCX) [file pone.0185534.s002.docx]

**S1 File. Data collection and analysis.** Further details of data collection and analysis methods.

*Data collection protocols*

Before the observation sessions started, three observers conducted a pilot study within one session to test and confirm the methods. Data recording sheets were provided, but observers were encouraged to record data by any method that was most efficient and would not draw attention to the study – for example in their notebook or on mobile phone. After describing the methods to the team, each observer was encouraged to conduct their own practice session, before commencing the data-collection sessions. There were several opportunities for observers to meet with the organisers in order to clarify the methods, provide feedback and submit data. To avoid double counting of audience members who moved between sessions, all counts of audience numbers were a snapshot count of those present at the end of the first talk of the session.

*Validation of the data collection*

Six sessions were monitored independently by two observers to test the consistency of the results. The total number of people in the audience recorded by separate observers within each session were strongly correlated (r=0.77) and there was no evidence that a linear model through the points had an intercept different to zero (F=3.281, df=1, p=0.130) or a slope different to one (F=2.749, df=1, p=0.158). An equivalent comparison of the proportion of the audience that was female estimated by two independent observers in the same six sessions also revealed a strong correlation (r=0.83) and no evidence that a linear model had an intercept different to zero (F=0.696, df=1, p=0.442) or a slope different to one (F=0.289, df=1, p=0.614). These results suggest that counts of numbers in the audience from independent observers were closely aligned and can be used together in a model.

Across the six sessions, 48 questions were recorded and 47 of these were recorded by both the observers. All 47 questions had agreement about the gender of the person asking the question. There was less consistency in recording audience members with hands up. Across the 47 questions, 14 people were recorded with hands up but not selected to ask a question, but only 2 of these people were recorded by both independent observers. This suggests that recording people with hands up is considerably harder to achieve, due to the necessity to scan the room very rapidly. The data of gender of people with their hands raised were not used in the analysis. There were an additional 4 questions in a discussion session and only 1 of these was recorded by both observers, suggesting questions within a discussion session were harder to delimit.

For the six sessions with double counts, the results of the audience gender proportions and seating locations were an average of the counts from the two independent observers. The results for the questions were from a randomly selected observer.
